# Occupational coping self-efficacy explains distress and well-being in nurses beyond psychosocial job characteristics

**DOI:** 10.3389/fpsyg.2015.01143

**Published:** 2015-08-06

**Authors:** Renato Pisanti, Margot van der Doef, Stan Maes, Caterina Lombardo, David Lazzari, Cristiano Violani

**Affiliations:** ^1^Faculty of Psychology, “Niccolò Cusano” University of RomeRome, Italy; ^2^Health, Medical and Neuropsychology Unit, Institute of Psychology, Leiden UniversityLeiden, Netherlands; ^3^Department of Psychology, University of Rome “Sapienza”Rome, Italy; ^4^Section of Clinical and Medical Psychology, “S. Maria” HospitalTerni, Italy

**Keywords:** professional burnout, psychological distress, job satisfaction, nurses, job demands control support model, occupational coping self-efficacy

## Abstract

**Aim:** The main purpose of the present study was to extend the Job Demand Control Support (JDCS) model analyzing the direct and interactive role of occupational coping self-efficacy (OCSE) beliefs.

**Background:** OCSE refers to an individual’s beliefs about their ability to cope with occupational stressors. The interplay between occupational stressors, job resources, and self-efficacy beliefs is poorly investigated. The present research attempts to address this gap.

**Design:** Cross-sectional survey.

**Method:** Questionnaire data from 1479 nurses (65% response) were analyzed. Hierarchical regression analyses were used to test the direct and moderating role of OCSE in conjunction with job demands (i.e., time pressure), and two job resources: job control (i.e., decision latitude and skill discretion) and social support (i.e., supervisor support and coworker support) in predicting psychological distress and well-being.

**Results:** Our findings indicated that high demands, low job control, and low social support additively predicted the distress/well-being outcomes (job satisfaction, emotional exhaustion, depersonalization, psychological distress, and somatic complaints). Beyond the main effects, no significant interactive effects of demands, control, and support were found. OCSE accounted for an additional 1–4% of the variance in the outcomes, after controlling for the JDCS variables. In addition, the results indicate that OCSE buffers the association between low job control and the distress dimensions emotional exhaustion, depersonalization, and psychological distress. Low control was detrimental only for nurses with low OCSE.

**Conclusion:** Our results suggest expanding the JDCS model incorporating individual characteristics such as OCSE beliefs, for predicting psychological distress and well-being. Limitations of the study and practical implications are discussed.

## Introduction

Research conducted in the health care sector in several countries suggests that nursing work has become increasingly stressful, with levels of psychological distress exceeding those of general population norms (Gelsema et al., 2007; Pisanti et al., 2011; Rudman et al., 2012). Moreover, some studies suggested that psychological distress and well-being of nurses could impact quality of care and patient safety (Van Bogaert et al., 2014; Panagopoulou et al., 2015; Welp et al., 2015).

In the last years a state of emergency within nursing professionals has been brought to light in Italy, regarding high turnover, high rate of retirement and contemporary low recruitment, so that the Italian heath care context is characterized by one of the lowest ratio nurses/per capita (6.0 active nurses per 1000) in Europe (Chaloff, 2008).

The main purpose of the present cross sectional study was to test how and to what extent an integrative theoretical framework – based on the interaction between occupational stressors, job resources, and coping self-efficacy – would explain various dimensions of occupational and general psychological well-being, in a sample of Italian nurses. The results may suggest different practical implications. Evidence for direct and/or moderating effects of occupational self-efficacy beliefs would lead to the recommendation to promote stress management training that focuses on how to cope more effectively in the health care context. On the other hand, if coping self-efficacy fails to moderate the impact of occupational stressors and does not have direct effects on well-being either, the focus should be on organizational interventions, aimed at improving the work environment.

### The Job Demands-Control- Support Model

To study the impact of occupational stressors on occupational and general psychological distress/well-being, the Job Demands Control- Support (JDCS) model is regarded as a useful conceptual framework (Karasek and Theorell, 1990). The original version of the model assumes two basic hypotheses of how two key work variables, job demands (e.g., time pressure) and job control (e.g., decision authority and skill discretion), may combine and lead to various well-being outcomes: (1) the strain hypothesis which assumes additive and interactive (synergistic or enhancing) effects of these work variables: high job demands precipitate job strain, as does low job control; (2) the buffer hypothesis: the resource job control has a moderating effect on the relationship between job demands and job strain. Later, social support from co-workers and supervisors was added to the model (Johnson and Hall, 1988) as a second job resource. Similar to job control, social support may influence distress and well-being via three pathways: additively, exacerbating and buffering the impact of high demands. This is reflected in the iso-strain hypothesis, stating additive and/or interactive -synergistic pattern effects of demands, control, and support; and the buffer hypothesis stating that job control and social support decrease the negative impact of high demands on well-being.

A number of reviews (van der Doef and Maes, 1999a; de Lange et al., 2003; Gelsema et al., 2007; Häusser et al., 2010) examined whether job demands, job control and social support combine additively or interactively to explain well-being outcomes. Overall, a general conclusion from these reviews is that the additive hypotheses received more support than the interactive hypotheses.

It is also increasingly recognized that individual variables such as locus of control, optimism, proactivity, coping and self-efficacy beliefs, affect, over and above the effects of JDCS variables, occupational strain and well-being through direct and moderating effects (Semmer and Meier, 2009; Rubino et al., 2012). All these constructs refer to the general tendencies by the individuals of interpreting the world, their relationship with it, and the possibilities to deal with it. For example, locus of control refers to the belief of the individuals that most general life outcomes are the result of their own actions (Rotter, 1990). Several studies have suggested that internal control beliefs (an individual’s beliefs in having personal control over their life) rather than external control beliefs (a person’s beliefs in forces outside their control) are crucial dimensions of adjustment and ability to handle stress in general and in one’s working life (e.g., Siu et al., 2002). These studies have found associations between locus of control and psychological distress/well-being outcomes. Likewise, high levels of optimism (Mäkikangas and Kinnunen, 2003), functional coping strategies (i.e., approach-oriented coping; de Rijk et al., 1998) and job self-efficacy (Jex and Bliese, 1999) showed to be associated with lower psychological distress and higher psychological well-being. As regards a moderating effect, it has been found that the hypothesized stress-buffering effects of job resources (job control and social support) on job demands to explain psychological distress and psychological well-being may only be found among individuals who report high scores on these dimensions, because they are more able to benefit from job resources (Parkes, 1991; de Rijk et al., 1998; van der Doef and Maes, 1999a; Kain and Jex, 2010). As such, job situations are not inherently stressful for all employees, and job resources do not seem to have the same beneficial moderating effects on the stressor-strain relationship for all employees. The current study focuses on employees’ occupational coping self-efficacy (OCSE) beliefs, an individual factor likely to play a direct and moderating role in the relationship between job characteristics and employee well-being. Also from a practical perspective, focusing on this specific individual factor is relevant, as coping self-efficacy beliefs are amenable to change and interventions to raise these beliefs are available (Bandura, 1997).

Finally, it has been suggested that the limited support for the buffer hypotheses of the JDCS model could be attributed to the use of general scales to assess the JDCS dimensions (de Lange et al., 2003; de Jonge et al., 2010). Occupation-specific measures, being more able to capture the relevant demands, control and support aspects of a job, might be required to adequately examine the moderating effect postulated by the JDCS model. Therefore, in the present study a measure developed with the specific purpose to assess nurses’ job characteristics was used.

### Occupational Coping Self-Efficacy

One factor that has been shown to influence the response to negative events such as occupational stressors is self-efficacy (Bandura, 1997). The general construct of self-efficacy refers to the belief that an individual has in their ability to execute a task and thus to obtain the desired outcome (Bandura, 1997). Self-efficacy expectancies can influence the level and persistence of efforts made to adopt a behavior: an individual will be most likely to adopt a behavior if they perceive that they are capable of adopting the behavior, and also if they believe that the outcome of such behavior will have a particular desired effect. As suggested by Maddux (2002, p. 278) “Self-efficacy is defined and measured not as a trait but as beliefs about the ability to coordinate skills and abilities to attain desired goals in particular domains and circumstances.” Moreover, several authors have also argued that a continuum exists which varies from generalized self-efficacy (Schwarzer et al., 1999) to more specific types of self-efficacy. According to the literature, many studies have developed and made use of “general” self-efficacy measures, but “they have not been as useful as more specific self-efficacy measures in predicting what people will do under more specific circumstances” (Maddux, 2002, pp. 278–279). Several occupational stress studies have considered the job self-efficacy construct, that concerns the employees’ beliefs of capability to fulfill their job tasks adequately (Jex and Bliese, 1999; Jimmieson, 2000; Salanova et al., 2002; Borgogni et al., 2013; Mazzetti et al., 2014) and have shown significant associations with psychological distress/well-being variables, especially with professional functioning dimensions (e.g., personal accomplishment, aspiration, and competence at work; Evers et al., 2002; Salanova et al., 2002; Borgogni et al., 2013). The focus of this study is on a specific type of job self-efficacy: OCSE. OCSE beliefs involve an individual’s beliefs about their ability to cope with occupational stressors. Despite other constructs of job self-efficacy, OCSE is characterized by two issues. First of all, it is more specific in the sense that it focuses on an individual’s beliefs about *their ability* to deal with situational stressors (Bandura et al., 1985). Secondly, it refers to these coping abilities in relation to the specific *stressors* one encounters in the job, such as work overload and interpersonal conflicts (with e.g., coworkers, patients). As such, OCSE can be distinguished from more general job related self-efficacy that refers to employees’ beliefs in their abilities to perform adequately and execute their work tasks, and follows the suggestion that the assessment of self-efficacy beliefs should be tailored to the particular domain of functioning that is the object of interest (Salanova et al., 2002).

Outside the literature on occupational stress, several studies have emphasized the central role of coping self-efficacy (CSE) in individuals for recovering from traumatic, stressful, and threatening events. High CSE has been related with a better management of stressful life changes and events such as aging (Kraaij et al., 2002), chronic disease (HIV-seropositive, Chesney et al., 2006), natural disaster (Benight and Bandura, 2004) and physical assault (Ozer and Bandura, 1990).

In general, these findings indicated that positive self evaluative beliefs, such as CSE, have direct effects on distress/well-being outcomes, over and above the influence of demanding situations. Individuals with high levels of CSE are prone to adaptively approaching environmental demands, viewing them as challenging and as positive experiences and promoting behavioral and cognitive adjustments. By contrast, individuals with low levels of CSE are more likely to appraise the same demanding tasks as stressful and are more likely to invest more energy to handle the increasing emotional distress (Bandura, 1997). On this basis, one can assume that OCSE is positively associated with employee well-being. However, to our knowledge, no published studies have looked at the relationship between OCSE and employee distress/well-being. Only Schwarzer (2003), in a theoretical paper, argued that the stronger one’s perceived efficacy to cope with occupational stressors, the more proactive and persistent one’s efforts will be in dealing with the demands (*proactive coping*).

In the context of the JDC model, however, a limited number of studies have examined the role of job-related self-efficacy beliefs, and we will draw on this research to formulate our hypotheses concerning the direct and moderating role of OCSE. Schaubroeck and colleagues found that only among workers with high job related self-efficacy job control moderated the negative effect of high demands on blood pressure (Schaubroeck and Merritt, 1997) and on chronic symptoms of upper respiratory infections (Schaubroeck et al., 2001). For those low in self-efficacy, high job control combined with high job demands was associated with negative health consequences, a finding that is in contrast with predictions derived from the JDC model. A more recent study (Meier et al., 2008) tested the three-way interaction hypothesis (Demand × Control × Self-efficacy) in a sample of 96 service employees, with affective strain and musculoskeletal pain as dependent variables. The interaction was significant only with regard to affective strain. Similar to Schaubroeck and Merrit’s findings, the predicted buffering effect of high job control on the impact of job demands was found for those high on self-efficacy, whereas high job control tended to increase the affective strain attributable to job demands for those low in self-efficacy. Likewise, in a longitudinal study conducted in a sample of 100 customer service representatives, Jimmieson (2000) only found evidence for the buffer role of job control on the impact of role conflict on depersonalization among individuals with high job self-efficacy. For the other three dependent variables of the study (psychological well-being, job satisfaction, and somatic health), however, no moderating effect of self-efficacy was found. Finally, Salanova et al. (2002) attempted to extend the JDC model, examining two alternative dimensions of self-efficacy, generalized self-efficacy, and task-specific (computer) self-efficacy, in a group of 405 workers using information technology in their jobs. Only task-specific self-efficacy was directly associated with the burnout dimensions regardless of the levels of the JDC variables. Furthermore, they found a three-way interaction with both self-efficacy measures in predicting the burnout dimensions exhaustion and cynicism. However, only for task-specific self-efficacy were the results in line with the prediction: job control buffered the effects of job demands on burnout among workers with high task specific self-efficacy, and it had stress-enhancing effects among those with low task specific self-efficacy.

Taken together, the results of these studies suggest that job control buffers the negative impact of job demands mainly for employees with high job-related self-efficacy. For efficacious employees, facing demanding situations with a lack of job control could be particularly harmful. On the other hand, high job control may enhance distress in low self-efficacious employees, as such a job context forces them to assume control that they feel unprepared to use (Litt, 1988). We expect the moderating effect of job control on the stressor-strain relationship to be similarly dependent on the employees’ OCSE.

So far, research on self-efficacy in the occupational context has mostly neglected social support, which is surprising from a JDCS perspective. Social support may be regarded as a workplace resource provided by others, as coping assistance or as an exchange of resources. In line with the findings concerning job control, one could postulate that the extent to which the job resource social support acts as a buffer is dependent on the employees’ self-efficacy beliefs. The single study that addressed this issue found that social support buffered the stressor-strain relationship in employees with high self-efficacy (Stetz et al., 2006). However, in employees with low self-efficacy a reverse buffering effect was evident, suggesting that high support increases negative reactions to the stressor in low self-efficacious employees. For this latter group of employees, high social support might instigate additional pressure and underscore their idea that they are not capable to handle the situation by themselves (Stetz et al., 2006). These results are in line with the enabling and the cultivation hypothesis (Schwarzer and Knoll, 2007) that explain what happens with perceptions of demands and support, among workers with different levels of self-efficacy. For the enabling hypothesis, social support may facilitate employee adaptation by enabling worker’s adaptive capabilities to face occupational stressors (providing opportunities to engage in vicarious experience in dealing with stressors); the cultivation hypothesis takes into account the reverse pathway: the relationship between receiving support and well-being seems to be amplified by self-efficacy beliefs because efficacious employees are more prone to invest efforts to improve and cultivate their social networks (e.g., “They go out and make social contacts, they take action to maintain valuable social relationships” Schwarzer and Knoll, 2007, p. 246) than employees with low self-efficacy beliefs. On this basis, we can hypothesize that low perceived support makes a stressful situation worse for efficacious workers, because it sends “negative relational feedback” which is more threatening for the individuals with high self-efficacy beliefs than for their counterparts with low levels of self-efficacy. On the other hand, when low self-efficacy individuals perceive high levels of social support, they may appraise themselves as not capable to deal with the situation by themselves. Thus, for low self-efficacy individuals, social support may strengthen (reverse buffer) the stressor-strain relationship.

### The Present Study

In order to examine employee psychological distress/well-being comprehensively (Van Horn et al., 2004), outcome variables from several distress/well-being dimensions were included in the present cross sectional study, namely indicators of burnout (affective, social, and professional dimensions), psychological distress, somatic complaints (affective and psychosomatic components), and job satisfaction (cognitive dimension).

Burnout can be described as a combination of emotional exhaustion, depersonalisation, and diminished personal accomplishment that may occur among individuals “who work with other people in some capacity” (Maslach, 1982). Professional burnout affects approximately 25% of nurses, but this percentage can rise to 64% in nurses who work in ward characterized by high affective strain and 39% in those with high cognitive strain (Estryn-Béhar et al., 1990).

Furthermore, we considered job satisfaction because previous studies (Lu et al., 2005) had identified it as a key factor in nurses’ recruitment and retention. Job satisfaction could be defined as “a positive (or negative) evaluative judgment one makes about one’s job or job situation” (Weiss, 2002, p. 175).

Finally, we considered two measures of distress: psychological distress and somatic complaints. Psychological distress, in this paper defined as anxiety (which includes tension, apprehension, and nervousness) and depression (namely symptoms of anhedonia, self-deprecation, and dysphoric mood), is a common complaint in nursing personnel (Eriksen et al., 2006). Somatic complaints involve symptoms caused by the perception of bodily dysfunction, such as headache and back pain. We considered somatic health complaints because in previous researches nurses, as health care workers, showed that somatic complaint levels are above average risk (Dollard et al., 2007).

On the basis of the JDCS model and the empirical research described, the following four hypotheses are examined:

H1 High job demands, low control and low social support are additively related to high emotional exhaustion and depersonalisation, low personal accomplishment, high psychological distress and somatic complaints, and low job satisfaction (additive hypothesis).
H2 In accordance with the interactive models, we hypothesize that low levels of job control and social support will enhance the detrimental effects of job demands on psychological distress and well-being (H2a, enhancing or synergistic pattern); and that high levels of job control and social support moderate the harmful associations between job demands and psychological distress and well-being (H2b, buffer hypothesis).
H3 Occupational coping self-efficacy accounts for additional variance in employee occupational and general psychological distress/well-being, after controlling for background variables and job dimensions. Higher levels of OCSE are associated with lower distress and higher well-being; in line with findings of previous mentioned studies (Evers et al., 2002; Salanova et al., 2002; Borgogni et al., 2013) we expected the strongest association with personal accomplishment.
H4 The buffering effect of control and/or social support on the association between demands and distress/well-being is only observed in individuals with higher levels of OCSE (H4a), conversely it is hypothesized that job control and/or social support act(s) as a stress exacerbator in individuals with lower levels of OCSE (H4b).

## Materials and Methods

### Sample and Procedure

Participants were nurses from nine different public Hospitals. Sampling was conducted to account for variances in the kind of health care organizations (i.e., General-, Academic-, High-Specialized-, Community- Hospital). The sample was drawn using a combined convenient and stratified sampling method. A list containing detailed information of all the health care organizations was conveniently obtained from two regions (Lazio and Umbria). Hospitals (Academic-, General-, High Specialized-, and Community-) were selected from the list paying attention to balancing the selected hospitals with regards to: (1) type of health care organization, (2) number of hospital beds per 1000 people, and (3) gender distribution in nursing staff. A total of nine health care organizations were drawn from the list meeting the sampling criteria. Different types of health care organizations were included and the number of hospital beds varied across the sample where, for example, General-Hospitals generally had a greater number of hospital beds than the High-Specialized-Hospitals. The number of males and females was in line with the gender distribution within the Italian health care context (77% female nurses). Within these hospitals a sample of nurses was approached for participation in the study. In order to equally represent nurses from the different organizations, the process recruitment took into account the size of the hospital. In hospitals employing <500 nurses, 75% of the nurses were approached, whereas in the larger hospitals (>500 nurses) 50% of the nurses were asked to participate. Using this procedure, a total of 2292 nurses across nine organizations were invited for the study, of which 1509 nurses agreed to participate. The number of participants ranged from 120 to 160 per hospital.

The voluntary nature of the study was emphasized. The research was approved by hospital ethics committees. Informed consent was obtained from all participants. After the exclusion of incomplete questionnaires, the final sample consisted of 1479 nurses (65% response rate). A comparison of the respondents to the non-respondents on gender and age showed that the 1479 nurses participating in the study were representative of those 2292 nurses who were asked to participate [with regard to gender: χ^2^(1) = 1.12; *p* > 0.05; age: *t*(2227) = -1.81; *p* > 0.05].

The mean age of the respondents was 39.2 years (SD = 8.4); 22.8% (*n* = 337) were men and 77.2% (*n* = 1142) were women. The mean tenure in the nursing profession was 15.5 years (SD = 9.2), 49% worked in medical and surgical ward, 20% worked in emergency ward, and 31.7% were community nurses.

### Measures

#### Psychosocial Job Characteristics

The psychosocial job characteristics were measured with three scales of the Italian version of the Leiden Quality of Work Life Questionnaire for Nurses (LQWLQ-N: Maes et al., 1999; Pisanti et al., 2009). The LQWLQ-N is an occupation-specific questionnaire based on the Leiden Quality of Work Questionnaire (van der Doef and Maes, 1999b) and has been applied in various studies on nursing populations (Gelsema et al., 2007; Pisanti, 2007; Adriaenssens et al., 2011). These three LQWLQ-N scales provide an occupation-specific measurement corresponding closely to the original operationalisation of job demands, control, and social support in the Job Content Questionnaire (JCQ; Karasek, 1985). Job demands were measured with one scale (work and time pressure: four items; e.g., “I must care for too many patients at once”). Control was assessed using a composite scale of skill discretion (four items; e.g., “My work is varied.”) and decision authority (four items; e.g., “I can decide for myself when to carry out patient-related tasks and when to carry out non-patient-related tasks.”). According to Karasek and Theorell (1990) skill discretion and decision authority are theoretically and empirically closely related and therefore often combined in one scale. A composite measure is frequently used in research on the JDCS model, also in studies on nurses (e.g., Bourbonnais et al., 1998; Bakker et al., 2005). Social support was assessed using a composite scale of social support from supervisor (six items; e.g., “I can count on the support of my direct supervisor when I face a problem at work.”) and social support from co-workers (six items; e.g., “The nurses in my department work well together.”). Responses are measured on a four-point scale ranging from 1 (*totally disagree*) to 4 (*totally agree*). To analyze whether these three psychosocial job dimensions represented distinct constructs, we carried out confirmatory factor analyses. In a model with three factors (job demands, job control, and social support) with all items loading on their respective factors, χ^2^ = 1374.39, *df* = 214, CFI = 0.94, RMSEA = 0.06, 90% RMSEA CI = 0.06–0.06, all factor loading were significant. Importantly, this three-factor model (with job control and social support hypothesized as inter-correlated) fit the data better than the best-fitting two-factor model with job control and social support loading on one common factor, χ^2^ = 3919.13, *df* = 215, CFI = 0.80, RMSEA = 0.11, 90% RMSEA CI = 0.10–0.11; Δχ^2^(1) = 2544.74, *p* < 0.001, and a one-factor model χ^2^ = 5064.822, *df* = 215, CFI = 0.74, RMSEA = 0.12, 90% RMSEA CI = 0.12–0.13; Δχ^2^(1) = 3690.43, *p* < 0.001.

#### Occupational Coping Self-Efficacy

The specific OCSE beliefs for nurses were measured using the Occupational Coping Self-efficacy scale for Nurses (OCSE-N). A more detailed description of its development, psychometric qualities and validation is described elsewhere (Pisanti et al., 2008). The OCSE-N consists of nine items with a five-point Likert type scale (1 ‘*not at all easy to cope with’* to 5 ‘*extremely easy to cope with*’). Instructions were given as follows: ‘the following statements describe occupational stressful situations which nurses may cope more or less easily with. For each situation, please rate how confident you feel you can easily cope with it’ (e.g., “Doing a lot of tasks at the same time”; “Relational difficulties with colleagues”). To examine the factor structure of the OCSE-N we tested three theoretical models by calculating first- and second-order confirmatory factor analyses. Model 1 defined one primary factor with loadings on all nine observed items. Model 2 consisted of two correlated primary factors corresponding to the two theoretical dimensions ‘coping Self-efficacy to cope with the occupational burden’ and ‘coping Self-efficacy to cope with the relational burden’ identified in the original paper (Pisanti et al., 2008). Model 3 consisted of the two primary factors and one second-order factor underlying the primary factors. Model 1 did not provide acceptable fit to the data, χ^2^ = 552.64, *df* = 25, CFI = 0.86, RMSEA = 0.12, 90% RMSEA CI = 0.11–0.13. However, Models 2 and 3 provided the same better fit than the one-factor model, χ^2^ = 218.56, *df* = 24, CFI = 0.95, RMSEA = 0.07, 90% RMSEA CI = 0.06–0.08, Δχ^2^(1) = 334.08, *p* < 0.001). Given that the last two models are equivalent and the final choice to adopt the first order structure or the second order structure is based on theoretical reasons (Byrne, 2010), in the present study the self-efficacy constructs are hypothesized as hierarchical factorial structure (Bandura, 2006) thus the second-order model was of primary interest to examine relationships between a general perception of OCSE and the other constructs in the study.

#### Distress/Well-Being Outcomes

Two categories of outcomes were assessed: general and occupational distress/well-being. General distress outcomes were assessed with scales from the Italian version (Violani and Catani, 1995) of the Symptom Checklist (SCL-90; Derogatis, 1983): psychological distress (consisting of 10 items on anxiety, e.g., “feeling afraid,” and 16 items on depression, e.g., “feeling lethargic”) and somatization (12 items, e.g., “headache”). Respondents indicated to what extent they had experienced each symptom over the past week. Answers were provided on a five-point scale (1 = *not at all*; 5 = *very much*). Job satisfaction and burnout were assessed as indicators of occupational distress/well-being. Job satisfaction was operationalised with the six-item of LQWLQ-N scale (e.g., “I am satisfied with my job”). Burnout was assessed by the Italian version (Pisanti et al., 2013) of the Maslach Burnout Inventory Human Service Survey (MBI-HSS; Maslach et al., 1996) which contains the three subscales: emotional exhaustion (eight items; e.g., “I feel frustrated by my job”); depersonalisation (five items; e.g., “I don’t really care what happens to some patients”) and personal accomplishment (seven items; e.g., “I feel exhilarated after working closely with my patients”). Participants were asked to rate from 0 (“*never*”) to 6 (“*daily*”) how often they experienced feelings described in each of the 20 items.

To examine if the six constructs were distinct, we carried out confirmatory factor analysis. A six factor model made up by psychological distress, somatization, emotional exhaustion, depersonalization, personal accomplishment and job satisfaction, provided a better fit, χ^2^ = 6909.04, *df* = 1915, CFI = 0.90, RMSEA = 0.04, 90% RMSEA CI = 0.04–0.04 than the best-fitting five-factor model, χ^2^ = 7760.28, *df* = 1920, CFI = 0.86, RMSEA = 0.05, 90% RMSEA CI = 0.05–0.05, Δχ^2^(5) = 851.24, *p* < 0.001; the best fitting four-factor model (χ^2^ = 10101.33, *df* = 1923, CFI = 0.80, RMSEA = 0.05, 90% RMSEA CI = 0.05–0.05), Δχ^2^(8) = 3192.29, *p* < 0.001; the best fitting three-factor model, (χ^2^ = 11093.35, *df* = 1926, CFI = 0.78, RMSEA = 0.06, 90% RMSEA CI = 0.06–0.06), Δχ^2^(11) = 4184.30, *p* < 0.001, with distress variables loading on one common factor; the best fitting two-factor model (χ^2^ = 14760.18, *df* = 1927, CFI = 0.69, RMSEA = 0.07, 90% RMSEA CI = 0.07–0.07, Δχ^2^(12) = 7851.136, *p* < 0.001) with distress variables loading on one common factor, and well-being variables loading on another common factor; and finally with one factor model, χ^2^ = 17039.14, *df* = 1928, CFI = 0.64, RMSEA = 0.07, 90% RMSEA CI = 0.07–0.07, Δχ^2^(13) = 10130.01, *p* < 0.001.

#### Data Analysis

Cronbach’s alphas, descriptive statistics and Pearson’s correlations were assessed. Data screening showed that, taking into account the large sample size, the assumptions of normality were not severely violated (-1.0 < skewness < 1.5; -0.4 < kurtosis < 2.5; West et al., 1995; Field, 2013).

The hypotheses of the study were tested in a series of hierarchical regression analyses.

Firstly, we controlled for the variables gender, age, organization, and the type of ward; because these background variables were correlated with both predictors and outcomes under study. Given that “organization” and “type of ward” are categorical variables, we adopted a dummy coding in the regression analyses. To test the hypotheses of the JDCS model, in the second block we entered the main effects of job demands, control and social support; subsequently, the two way (third block) and three way interactions (fourth block) of these JDCS variables were included. Next, to examine the influence of the individual factor OCSE, the main effect of OCSE was entered (fifth block); followed by the two way (sixth block), three way (seventh block) and four way (eighth block) interactive terms of OCSE with the JDCS variables. In the final analyses, a more parsimonious model was examined, including all main effects, the significant interactions, and those non-significant interactions that need to be included in the model in order to adequately test the higher order interactions (Cohen et al., 2003).

In all regression analyses the JDCS dimensions and OCSE were standardized to avoid multicolinearity that might otherwise result from the use of multiplicative terms (Cohen et al., 2003). Six multivariate outliers were identified using studentized residuals (Cohen et al., 2003), but kept in the analyses, as their Cook’s distance values indicated that they did not affect the final model (Field, 2013).

To examine the nature of the significant multiplicative interaction terms, they were graphically displayed according to the method proposed by Cohen et al. (2003). Values of the predictor variables were represented at one standard deviation below and one standard deviation above the mean. Regression lines were then estimated by entering these values in the regression equation.

## Results

The zero-order correlations of the variables, and their mean, standard deviation, and reliabilities (Cronbach’s α) are presented in **Table 1**. All scales measuring the study variables displayed acceptable to good reliability (alpha coefficients ranged from 0.70 to 0.94).

**Table 1:** Mean (M), standard deviation (SD), internal consistencies (Cronbach’s α), and zero-order correlation of the study variables (*N* = 1479).

Variable	*M*	SD	α	1	2	3	4	5	6	7	8	9	10	11
**Demographic variables** (1) Gender^a^ (2) Age **Job conditions** (3) Demands (4) Control (5) Social support **Personal variable** (6) Occupational coping Self-efficacy **Distress/well-being outcomes** (7) Emotional exhaustion (8) Depersonalization (9) Personal accomplishment (10) Psychological distress (11) Somatic complaints (12) Job satisfaction	— 39.2 2.9 2.8 2.8 3.0 2.2 0.9 4.5 1.8 2.1 2.4	— 8.4 0.7 0.6 0.6 0.7 1.3 5.0 1.1 0.6 0.7 0.5	— — 0.74 0.74 0.87 0.83 0.89 0.70 0.85 0.94 0.84 0.72	— 0.01 0.01 0.02 -0.00 -0.00 0.05 -0.11∗∗∗ 0.02 0.17∗∗∗ 0.15∗∗∗ -0.01	— — -0.03 -0.01 -0.00 0.17∗∗∗ 0.05∗ -0.04 0.13∗∗∗ 0.10∗∗ 0.09∗∗ 0.03	— — — -0.05 -0.05 -0.12∗∗∗ 0.19∗∗∗ 0.11∗∗∗ -0.13∗∗∗ 0.12∗∗∗ 0.17∗∗∗ -0.20∗∗∗	— — — — 0.44∗∗∗ 0.28∗∗∗ -0.22∗∗∗ -0.18∗∗∗ 0.25∗∗∗ -0.18∗∗∗ -0.15∗∗∗ 0.42∗∗∗	— — — — — 0.34∗∗∗ -0.26∗∗∗ -0.19∗∗∗ 0.19∗∗∗ -0.19∗∗∗ -0.22∗∗∗ 0.44∗∗∗	— — — — — — -0.30∗∗∗ -0.25∗∗∗ 0.22∗∗∗ -0.23∗∗∗ -0.22∗∗∗ 0.35∗∗∗	— — — — — — — 0.39∗∗∗ -0.19∗∗∗ 0.52∗∗∗ 0.51∗∗∗ -0.44∗∗∗	— — — — — — — — -0.33∗∗∗ 0.31∗∗∗ 0.19∗∗∗ -0.23∗∗∗	— — — — — — — — — -0.22∗∗∗ -0.14∗∗∗ 0.28∗∗∗	— — — — — — — — — — 0.65∗∗∗ -0.26∗∗∗	— — — — — — — — — — — -0.26∗∗∗

*^a^Male = 1; Female = 2.*

*∗*p* < 0.05; ^∗∗^*p* < 0.01; ^∗∗∗^*p* < 0.001.*

The correlations between the JDCS variables and the dependent variables were all significant and in the expected direction. OCSE was associated both with the JDCS variables and all dimensions of distress/well-being. More specifically, higher OCSE was associated with higher job control, social support, personal accomplishment and job satisfaction, and with lower job demands, emotional exhaustion, depersonalization, psychological distress and somatic complaints.

### Testing the Additive and Interactive Effects of the JDCS Model

**Table 2** presents the results of the hierarchical regression analyses in which burnout components, job satisfaction, psychological distress, and somatic complaints were regressed on the psychosocial job characteristics and OCSE.

**Table 2:** Distress/well-being outcomes regressed on the JDCS variables, Occupational Coping Self-efficacy, and their interactions.

Predictors	Job satisfaction	Emotional exhaustion	Depersonalization	Personal accomplishment	Somatic complaints	Psychological distress
^a^Block 1 Δ*R*^2^	0.03^∗∗^	0.05^∗∗∗^	0.05^∗∗∗^	0.14^∗∗∗^	0.05^∗∗∗^	0.05^∗∗∗^
Demands Control Social support	-0.14^∗∗∗^ 0.32^∗∗∗^ 0.28^∗∗∗^	0.20^∗∗∗^ -0.14^∗∗∗^ -0.19^∗∗∗^	0.07^∗∗^ -0.13^∗∗∗^ -0.11^∗∗∗^	-0.01 0.22^∗∗∗^ 0.10^∗∗^	0.19^∗∗∗^ -0.07∗ -0.15^∗∗∗^	0.10^∗∗∗^ -0.15^∗∗∗^ -0.12^∗∗∗^

Block 2 Δ*R*^2^	0.28^∗∗∗^	0.10^∗∗∗^	0.06^∗∗∗^	0.09^∗∗∗^	0.07^∗∗∗^	0.07^∗∗∗^

Demands Control Social support Demands × Control Demands × Social Support Control × Social Support	-0.14^∗∗∗^ 0.31^∗∗∗^ 0.28^∗∗∗^ 0.02 0.01 -0.01	0.20^∗∗∗^ -0.13^∗∗∗^ -0.19^∗∗∗^ -0.01 0.02 0.01	0.07^∗∗^ -0.12^∗∗∗^ -0.10^∗∗∗^ 0.03 0.01 0.02	0.00 0.22^∗∗∗^ 0.10^∗∗^ 0.00 -0.01 0.00	0.18^∗∗∗^ -0.07∗ -0.13^∗∗∗^ 0.02 0.01 0.01	0.10^∗∗∗^ -0.15^∗∗∗^ -0.10^∗∗∗^ 0.01 0.00 0.01

Block 3 Δ*R*^2^	0.00	0.00	0.00	0.00	0.00	0.00

Demands Control Social support Demands × Control Demands × Social support Control × Social support Demands × Control × Social Support	-0.14^∗∗∗^ 0.31^∗∗∗^ 0.27^∗∗∗^ 0.02 0.00 0.00 0.00	0.20^∗∗∗^ -0.12^∗∗∗^ -0.19^∗∗∗^ -0.01 0.01 0.01 0.01	0.07^∗∗^ -0.12^∗∗∗^ -0.10^∗∗∗^ 0.02 0.01 0.01 -0.01	0.00 0.21^∗∗∗^ 0.10^∗∗^ 0.00 -0.01 0.00 0.00	0.18^∗∗∗^ -0.06∗ -0.13^∗∗∗^ 0.01 0.00 0.01 0.02	0.10^∗∗∗^ -0.15^∗∗∗^ -0.08^∗∗^ 0.01 0.00 0.01 0.02

Block 4 Δ*R*^2^	0.00	0.00	0.00	0.00	0.00	0.00

Demands Control Social support Demands × Control Demands × Social Support Control × Social Support Demands × Control × Social Support OCSE	-0.15^∗∗∗^ 0.27^∗∗∗^ 0.25^∗∗∗^ 0.02 0.01 -0.01 0.01 0.19^∗∗∗^	0.20^∗∗∗^ -0.10^∗∗^ -0.10^∗∗^ -0.01 0.00 0.01 0.00 -0.26^∗∗∗^	0.07^∗∗^ -0.08^∗∗^ -0.07∗ 0.01 0.00 0.01 0.00 -0.17^∗∗∗^	-0.00 0.20^∗∗∗^ 0.07^∗∗^ -0.01 0.00 0.01 -0.01 0.13^∗∗∗^	0.16^∗∗∗^ -0.05 -0.08^∗∗^ 0.00 0.01 0.01 0.00 -0.22^∗∗∗^	0.09^∗∗∗^ -0.13^∗∗∗^ -0.05∗ -0.01 0.00 0.01 0.00 -0.20^∗∗∗^

^b^Block 5 Δ*R*^2^	0.03^∗∗∗^	0.04^∗∗∗^	0.03^∗∗∗^	0.01^∗∗∗^	0.03^∗∗∗^	0.03^∗∗∗^

Demands Control Social support Demands × Control Demands × Social Support Control × Social Support Demands × Control × Social Support OCSE Demands × OCSE Control × OCSE Social Support × OCSE	-0.14^∗∗∗^ 0.27^∗∗∗^ 0.24^∗∗∗^ 0.01 0.01 -0.01 0.01 0.18^∗∗∗^ 0.02 0.00 0.01	0.19^∗∗∗^ -0.09^∗∗^ -0.09^∗∗^ -0.01 0.00 0.01 0.00 -0.29^∗∗∗^ -0.01 0.15^∗∗∗^ 0.02	0.07^∗∗^ -0.07^∗∗^ -0.07^∗∗^ -0.01 0.00 0.01 0.00 -0.20^∗∗∗^ 0.00 0.08∗ -0.00	-0.00 0.20^∗∗∗^ 0.07^∗∗^ -0.01 0.00 0.01 0.00 0.13^∗∗∗^ 0.01 0.01 0.00	0.18^∗∗∗^ -.03 -0.10^∗∗^ -0.01 0.00 0.01 0.00 -0.22^∗∗∗^ 0.00 -0.00 0.01	0.09^∗∗∗^ -0.11^∗∗∗^ -0.06∗ -0.01 0.00 0.01 0.00 -0.22^∗∗∗^ -0.01 0.07∗ -0.01

Block 6 Δ*R*^2^	0.00	0.02^∗∗∗^	0.01∗	0.00	0.00	0.01∗

Demands	-0.14^∗∗∗^	0.19^∗∗∗^	0.07^∗∗^	-0.00	0.18^∗∗∗^	0.09^∗∗∗^
Control	0.26^∗∗∗^	-0.11^∗∗∗^	-0.07^∗∗^	0.20^∗∗∗^	-0.03	-0.11^∗∗∗^
Social support	0.25^∗∗∗^	-0.11^∗∗^	-0.07∗	0.07^∗∗^	-0.10^∗∗^	-0.07∗
Demands × Control	0.00	0.01	0.00	-0.01	0.00	-0.02
Demands × Social Support	0.01	0.00	0.01	0.00	0.01	0.01
Control × Social Support	-0.02	0.01	-0.02	0.01	-0.01	-0.02
Demands × Control × Social Support	0.00	0.01	0.01	0.02	0.00	0.00
OCSE	0.18^∗∗∗^	-0.31^∗∗∗^	-0.20^∗∗∗^	0.13^∗∗∗^	-0.22^∗∗∗^	-0.22^∗∗∗^
Demands × OCSE	0.01	-0.01	0.00	0.00	0.01	0.00
Control × OCSE	0.00	0.14^∗∗∗^	0.08∗	0.01	0.00	0.07∗
Social Support × OCSE	0.00	-0.04	-0.00	0.00	0.01	-0.01
Demands × Control × OCSE	-0.01	0.00	0.01	0.01	0.00	0.01
Demands × Social Support × OCSE	0.00	0.01	0.00	0.00	-0.01	0.00
Control × Social Support × OCSE	-0.01	0.05∗	0.00	0.00	-0.01	0.02
Block 7 Δ*R*^2^	0.00	0.01∗	0.00	0.00	0.00	0.00
*R*^2^ final model (Adj *R*^2^)	0.34^∗∗∗^ (0.33)	0.23^∗∗∗^ (0.22)	0.15^∗∗∗^ (0.13)	0.25^∗∗∗^ (0.24)	0.16^∗∗∗^ (0.15)	0.17^∗∗∗^ (0.16)

*The unstandardized regression coefficients (B’s) are reported.*

*∗*p* < 0.05; ^∗∗^*p* < 0.01; ^∗∗∗^*p* < 0.001.*

*OCSE = Occupational Coping Self-Efficacy, Block *n* Δ*R*^2^: *R* Square Change.*

*^a^In the block 1 were included the results of the gender and the dummy variables (public health care organizations and type of ward). Due to a lack of space we omitted the Beta’ s for these results.*

*^b^The four way interaction Demands × Control × Social Support × OCSE were consistently non-significant and therefore omitted in the final analyses.*

In line with the iso-strain hypothesis (Hypothesis 1), the analyses showed consistent additive effects of the psychosocial work dimensions on all outcomes, except in the case of personal accomplishment. Higher job demands, lower control and lower support were associated with higher levels of emotional exhaustion [*F change* (3,1268) = 52.3, *p* < 0.001, Δ*R*^2^ = 10%], depersonalization [*F change* (3,1302) = 27.1; *p* < 0.001, Δ*R*^2^ = 6%], somatic complaints [*F change* (3,1271) = 33.4, *p* < 0.001, Δ*R*^2^ = 7%], and psychological distress [*F change* (3,1311) = 33.2, *p* < 0.001, Δ*R*^2^ = 7%]. Furthermore, lower levels of job demands, higher levels of control and higher levels of social support were related to higher levels of job satisfaction [*F change* (3,1297) = 175.9, *p* < 0.001, Δ*R*^2^ = 28%]. Finally, higher levels of both job resources (job control and social support) were associated with higher levels of personal accomplishment [*F change* (3,1251) = 48.1, *p* < 0.001, Δ*R*^2^ = 9%].

With regard to the second hypothesis focusing on the moderating effects of control and social support, analyses did not show any significant interaction effect.

Furthermore, no evidence for the hypothesized three-way interaction of demands, control, and support was found. Thus we can conclude that Hypothesis 2 did not receive any support.

### The Role of Occupational Coping Self-Efficacy

In line with Hypothesis 3, OCSE explained a significant proportion of the variance in all outcomes beyond the background and JDCS variables (see **Table 2**). More specifically, OCSE explained the highest additional variance, 4%, for emotional exhaustion [*F change* (1,1263) = 68.2, *p* < 0.001], followed by 3% for psychological distress [*F change* (1,1306) = 50.4, *p* < 0.001], somatic complaints [*F change* (1,1266) = 49.4, *p* < 0.001], depersonalization [*F change* (1,1297) = 39.2, *p* < 0.001], and job satisfaction [*F change* (1,1292) = 55.2, *p* < 0.001]; and 1% for personal accomplishment [*F change* (1,1246) = 25.2, *p* < 0.001]. As expected, lower OCSE was consistently associated with higher distress and lower well-being.

**FIGURE 1:**
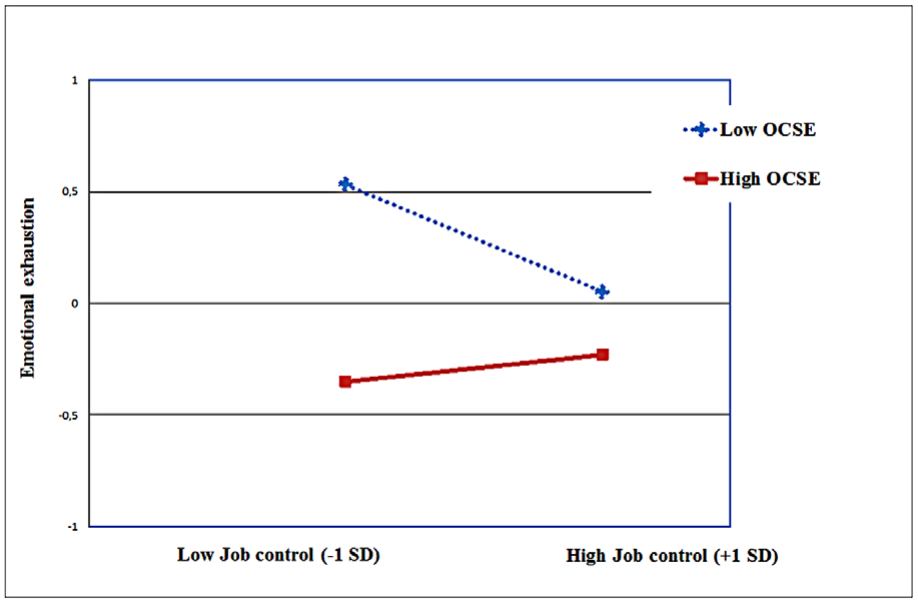
**Job control X OCSE, predicting emotional exhaustion**.

**FIGURE 2:**
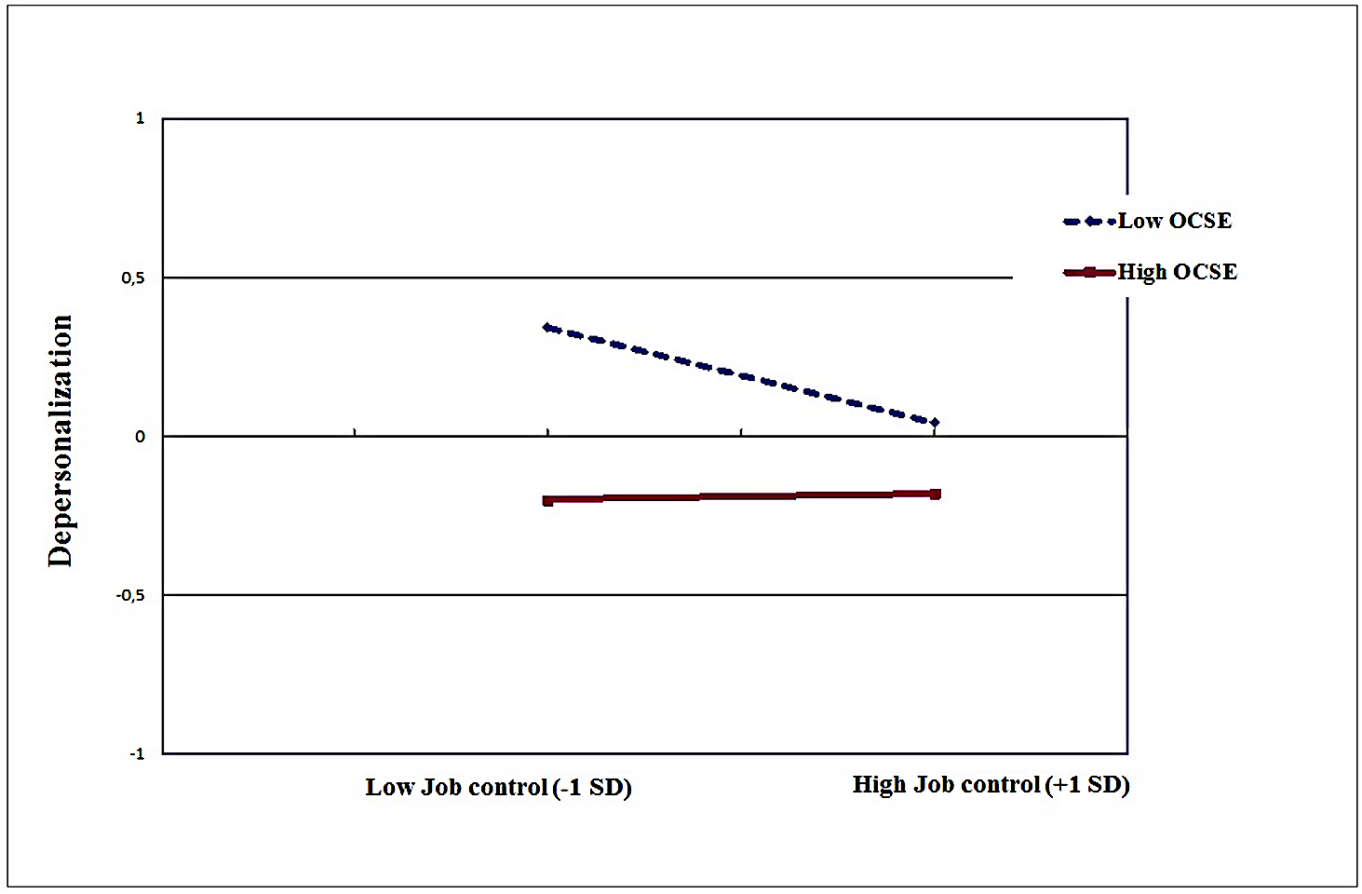
**Job control X OCSE, predicting depersonalization**.

**FIGURE 3:**
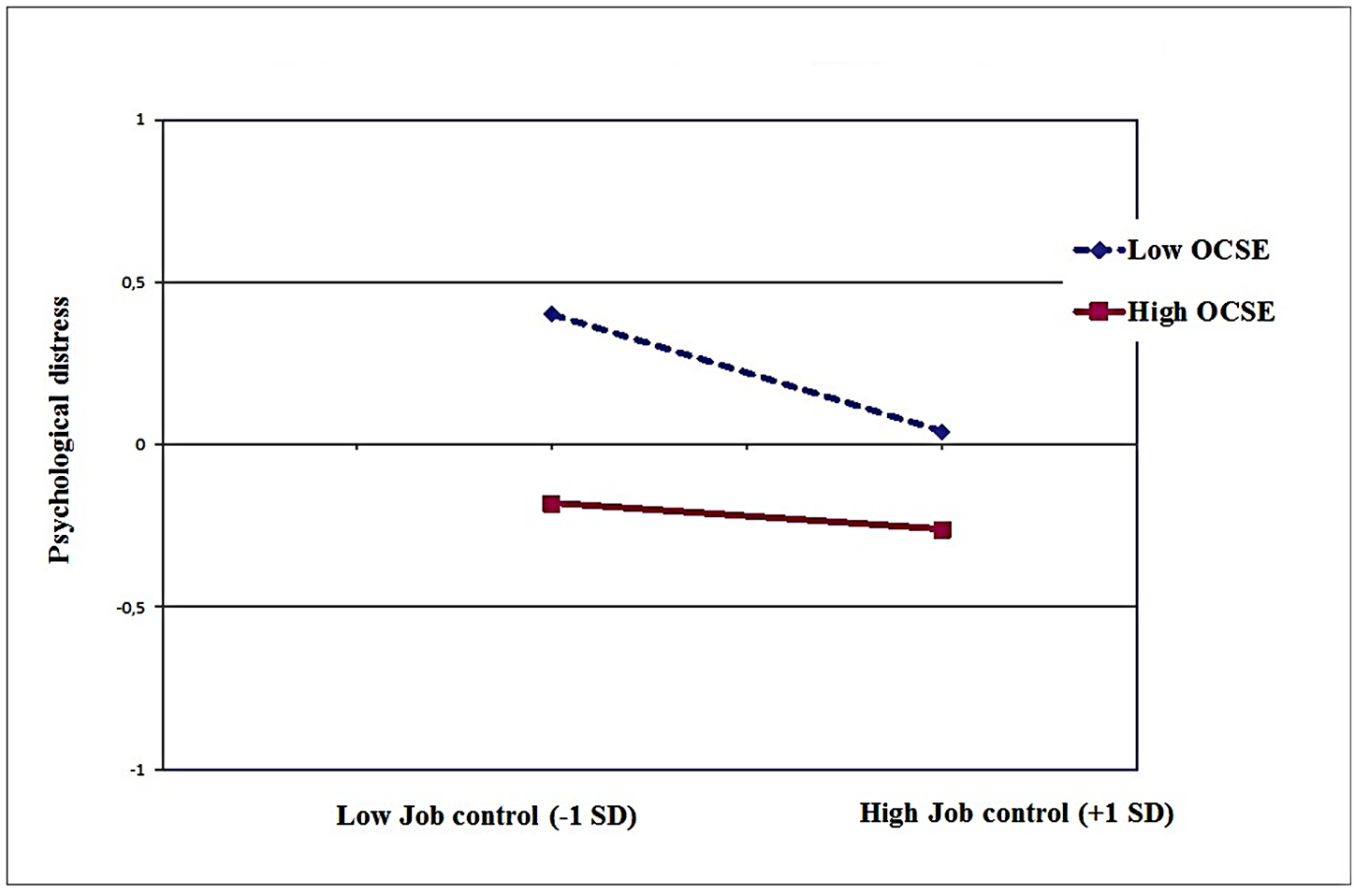
**Job control X OCSE, predicting psychological distress**.

As described in **Table 2**, the regression analyses yielded significant two way interactions between OCSE and job control in predicting emotional exhaustion (*B* = 0.15, *p* < 0.001), depersonalization (*B* = 0.08, *p* < 0.01), and psychological distress (*B* = 0.07, *p* < 0.01). The interactions accounted for 2% of the additional variance in the case of emotional exhaustion; and 1% in the other instances. As depicted in the **Figures 1–3**, the nature of the interaction is similar. Simple slopes analysis (Dawson and Richter, 2006) indicated that (low) control had the strongest influence on emotional exhaustion when perceived OCSE was low (*B* = -0.24, *p* < 0.001). The slope for high OCSE was not significant (*B* = 0.02, *p* > 0.10). Similar results were obtained for depersonalization (slope for low OCSE: *B* = -0.16, *p* < 0.001; slope for high OCSE: *B* = -0.04, *p* > 0.10); and psychological distress (slope for low OCSE: *B* = -0.12, *p* < 0.001; slope for high OCSE: *B* = 0.01, *p* > 0.10).

**FIGURE 4:**
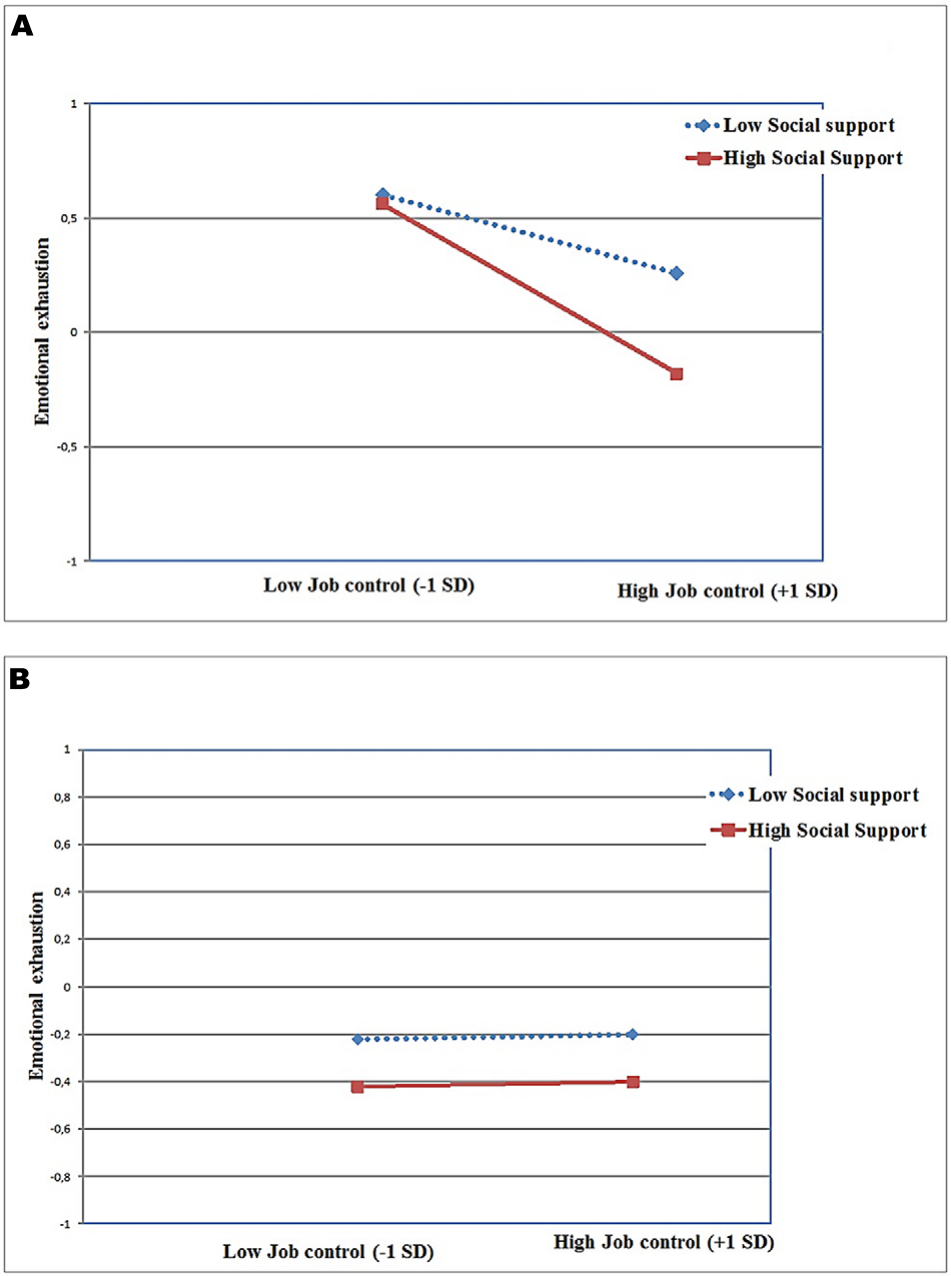
**(A)** Job control X Social support X OCSE predicting emotional exhaustion in low OCSE. **(B)** Job control X Social support X OCSE predicting emotional exhaustion in high OCSE.

Furthermore, a significant three way interaction of job control, social support and OCSE on emotional exhaustion (*B* = 0.05, *p* < 0.05) was found. This interaction is illustrated in **Figures 4A,B**, depicting the associations between demands, control, and emotional exhaustion separately for low OCSE (-1 SD) employees and high OCSE (+ 1 SD) employees. Inspecting the simple slopes indicated that nurses with high OCSE (**Figure 4B**) scored low on emotional exhaustion regardless of their levels of job control and social support (*t*-value for slope difference = 1.02; *p* > 0.10). For nurses with low OCSE (**Figure 4A**), high control was associated with lower emotional exhaustion; this relationship was stronger when these nurses also experienced high levels of social support (*t*-value for slope difference = -3.55; *p* < 0.001).

## Discussion

In the present study we hypothesized an integrative job stress model that included job demands and job resources in conjunction with self-efficacy beliefs in predicting psychological distress and well-being. We addressed a noteworthy limitation of the JDCS model (i.e., the use of general scales to assess the JDCS dimensions, de Lange et al., 2003; de Jonge et al., 2010) and we tested the role of OCSE as specified in Social Cognitive theory. This hypothesized integration is in line with recent theoretical frameworks, as the Job Demands Resources Model (Bakker et al., 2004) and the Demand-Control-Person model (Rubino et al., 2012). More specifically, we aimed to investigate the relationships between occupation-specific assessed psychosocial job dimensions and job-related and general distress/well-being in nurses, focusing on both the additive and interactive hypotheses of the Job Demands-Control-Support (JDCS) model; and on the direct and moderating role of an individual variable, OCSE, in this context.

First, with regard to our first hypothesis, we found support for the additive main effects of demands, control, and support across outcomes, except in the case of personal accomplishment. These findings are in line with previous research on nurses (e.g., Sundin et al., 2007). Also in line with previous research (e.g., Bakker et al., 2005; Rodwell et al., 2009) job satisfaction and personal accomplishment are more strongly related to the job resources control and support, whereas job demands and support are more strongly associated with emotional exhaustion, and somatic complaints. For depersonalization and psychological distress, demands, control, and support seem to play a more equal role. These findings support the notion that job demands and job resources exert differential effects on outcomes, possibly through respectively an energy-depleting process, affecting psychological distress dimensions (e.g., emotional exhaustion), and a motivational process, affecting well-being dimensions (e.g., job satisfaction; Bakker et al., 2004).

Beyond the main effects previously discussed, we did not find any significant interactive effects between demands, control, and support. Although we adopted a specific measurement of JDCS variables for nurses, Hypothesis 2 was not supported in our study. This finding is in line with Taris (2006), who concluded that full support for the buffer hypothesis was found in a small percentage of studies, little more than chance level. The available evidence suggests that the interactive effect is an exception rather than the rule.

As predicted in our third hypothesis, high levels of OCSE were consistently associated with higher well-being and lower distress. After taking into account the background and JDCS variables, OCSE explained 1–4% of additional variance in the six indicators of wellbeing/distress under study. All relationships were in the predicted direction: OCSE was negatively related to all distress variables (emotional exhaustion, depersonalization, psychological distress, and somatic complaints) and positively related to both positive outcomes (job satisfaction and personal accomplishment). Within a work context, only Salanova et al. (2002) found consistent associations between a specific measure of self-efficacy (Computer Self-efficacy) and burnout dimensions regardless of the levels of JDC variables. These and our findings lend support to the notion that it is important to measure self-efficacy related to the specific tasks employees have to deal with in their work context in order to gain insight into employee well-being and distress. Contrary to expectations, OCSE was consistently associated with all distress and well-being measures (affective, cognitive, social, professional, and psychosomatic dimensions), and least strongly to personal accomplishment. This lend support to the specific type of self-efficacy adopted in the present study. Individuals with higher levels of OCSE are more likely to interpret occupational stressors as challenging situations. As a result, they may be more likely to invest more effort to effectively deal with a less favorable work situation, thereby reducing the potential for development of negative outcomes, and maintaining and enhancing the positive effects of their job (Bandura, 1997). Probably, the strongest associations between professional and job self-efficacy measures with professional functioning dimensions (i.e., personal accomplishment) found in previous studies (Evers et al., 2002; Salanova et al., 2002; Borgogni et al., 2013), were due to the same level of specificity of both sets of indicators. We therefore suggest differentiating self-efficacy beliefs (OCSE and job self-efficacy) in future studies on occupational stress and well-being.

In Hypothesis 4 we postulated that OCSE would be a crucial individual factor on which the support for the moderating role of job control would be dependent. In our study, we found no evidence for that proposition; the relevant interaction effects involving demands, control and OCSE were all non-significant. Job control did not emerge as a moderator of the impact of job demands, not for the whole sample as mentioned previously, and neither for specific subgroups in terms of OCSE. However, we found evidence suggesting that OCSE buffers the negative impact of *lack of job control* on distress. In other words, especially for nurses with low OCSE lower job control was associated with higher levels of distress. This finding is quite in contrast to the expectation that for employees with low OCSE high job control would be stress-enhancing (see, e.g., Litt, 1988). An explanation could be that within this specific professional group of Italian nurses, job control levels are not so high that they become an additional burden for the low efficacious nurses (Pisanti et al., 2011). So, our study indicates that believing that a situation is uncontrollable does not always lead to an increase in distress. The appraisal of both external resources (job control) and internal coping resources (OCSE) as low seems to put employees at risk for burnout (emotional exhaustion and depersonalization) and psychological distress, regardless of their level of demands. Furthermore, the results suggest that OCSE as an internal resource can compensate the lack of external job resources, in this case job control. The lack of support for this interaction in the case of the positive dimensions in our study (personal accomplishment and job satisfaction) is in line with studies mentioned in the introduction (Jimmieson, 2000; Salanova et al., 2002) and might be due to a ceiling effect, since nurses perceiving high levels of job resources and OCSE already experience high levels of well-being (motivational process), which may preclude further increases in well-being through further compensation of OCSE. Another possible explanation could relate to the different processes underlying the two sets of variables (affective and social vs. cognitive and professional). Emotional exhaustion, depersonalization, and psychological distress can be considered as outcomes of depleting processes that involve, additively and interactively, psychosocial job dimensions, and individual variables such as self-efficacy beliefs. By contrast, job satisfaction and personal accomplishment can be considered as more “momentary” cognitive evaluations of the quality of one’s job and of one’s performance, where individual variables are additively involved and do not act as moderators on any psychosocial job dimension. Taken together, these findings underline the importance of considering how different forms of distress and well-being are associated with situational and individual factors (Taris and Schaufeli, 2015).

In addition, we found a significant three way interaction between OCSE, job control, and social support in predicting emotional exhaustion. High levels of OCSE are consistently associated with lower levels of emotional exhaustion regardless of the levels of support and control. Nurses with low levels of OCSE, however, seem to require high job control to attenuate emotional exhaustion – an effect that is strengthened when they perceive their working environment as supportive. In other words, nurses who lack both the internal resource of high OCSE and the external resources of high job control and social support experience the highest emotional exhaustion. Again, a finding in contrast with the notion that for employees with low OCSE, high job control would be stress-enhancing.

It should be noted that whereas OCSE appears to moderate the impact of the job resources, job demands are not involved in any of the significant interactions. This may be explained by the fact that whereas OCSE involves coping self-efficacy with regard to an array of stressors a nurse is likely to experience, the assessment of demands focuses mainly on work and time pressure. Although the latter is in line with the operationalization of the construct of demands of the JDCS model (Karasek, 1985), for future studies it would be recommendable to include other demanding aspects of the job (e.g., emotional demands; van Vegchel et al., 2004).

The interactive effects in the current study add, albeit significant, a limited proportion to the explained variance in the outcomes under study. This is generally the case in regression analyses, due to the amount of variance already explained by the main effects of the predictors. However, as indicated by, for instance, Wall et al. (1996), this does not indicate that the moderating effect has limited theoretical and practical implications. The variance explained in a subgroup can be quite large even when the overall effect is small (Cohen et al., 2003). In our study, for instance, for the *high OCSE* employees job control was not significantly associated with emotional exhaustion, depersonalization, and psychological distress. However, for employees with *low OCSE* lower levels of control were significantly associated with higher scores on these outcomes. For the subgroup of nurses with low OCSE (< -1.0 SD) low job control was a significant predictor for emotional exhaustion (*B* = -0.24, *p* < 0.001); for depersonalization (*B* = -0.16, *p* < 0.001); and for psychological distress (*B* = -0.12, *p* < 0.001) whereas for the subgroup of nurses with high OCSE (> +1.0 SD), job control failed to be associated with any outcome under study.

### Practical Implications

The most important implication of this study stems from the fact that in the explanation of employee burnout and well-being we found no support for interactive effects between job stressors and job resources, and limited support for interactive effects between job characteristics and OCSE beliefs. We found interactive effects between OCSE and job control in explaining emotional exhaustion, depersonalization, and psychological distress. The findings thus emphasize that both organizational and individual interventions are warranted in order to increase occupational well-being. Therefore, reducing job demands and fostering job resources by making changes in the job design (e.g., task enrichment, decentralization of decision authority), and the working environment (e.g., staffing, managerial style); seem promising initiatives, for these factors play an important role in the development of occupational well-being on the one hand and burnout symptoms and health complaints on the other hand. Furthermore, it seems worthwhile to implement stress management training that focuses on improving nurses’ beliefs in their OCSE, and on enhancing their potential to implement ways of coping that are appropriate to the circumstances. Coping self-efficacy beliefs could be improved through four processes: mastery experiences (e.g., sessions that provide experiences on how to handle situational stressors successfully), vicarious experience (e.g., providing analyses on how colleagues’ deal with stressful situations), verbal persuasion (e.g., instrumental and emotional support from more experienced and respected fellow nurses), and physiological states (e.g., analyses of feedbacks from physiological and emotional cues when dealing with situational stressors). Several authors (e.g., Bandura, 1997; Zimmerman, 2000) indicate that the most influential way to boost self-efficacy beliefs is by enabling “mastery experiences.” Mastery experiences workshops permit individuals to actively experience the positive effects associated with their actions; as a result their interpretations of these effects boost their efficacy beliefs. Success in handling occupational stressors increases self-efficacy, whereas failure could reduce it. Therefore, one could also focus on tools such as an after-event review (Ellis et al., 2010) to analyze the causes for success or failure in facing occupational stressors. Moreover, interventions adopting tools addressed to improve Emotional and Social Intelligence competencies (e.g., self-management, social awareness and relationship management behaviors) could stimulate a learning approach and engagement in mastery experiences in order to increase OCSE. Codier et al. (2011) have shown that this type of interventions implemented among nurse managers could also have positive effects on organizational outcomes, such as performance levels, team effectiveness, improved communication and positive conflict styles. However, future studies addressing effectiveness of these types of training in nurses and their consequences on coping self-efficacy are needed.

### Study Limitations and Suggestions for Future Research

The current study has some limitations that should be acknowledged. First, this study was focused on the nursing profession – a necessity to enable the use of occupation-specific measures of job characteristics and OCSE. However, this restriction to a single occupational group might hamper the generalization of the findings to other occupational groups. Second, given its cross-sectional design, this study does not provide possibilities for causal inferences regarding psychosocial characteristics, OCSE, and distress/wellbeing. As such, the possibility of reversed or reciprocal causality cannot be ruled out. A carefully designed longitudinal study, with appropriate time intervals (cf. Zapf et al., 1996) could provide further insight into the causal processes involved. Finally, our study relies on self-reported measures. As a consequence, common method variance may have inflated the relationships between the predictors and the outcomes (Podsakoff et al., 2003). However, the constructs did not show strong intercorrelations: their standardized coefficients varied between 0.12 and 0.48, implying that they are likely to assess separate, although related dimensions, rather than the same variable (see Chan, 2009). Moreover the correlations among variables could be inflated by some variables such as such as mood, expectations, previous experiences, or health (Rugulies, 2012). However, as suggested by several authors (Spector et al., 2000; Spector, 2006; Chan, 2009) this problem is probably overrated. For example, Spector et al. (2000) argued that removing the variance of negative affectivity could actually reduce some of the variance in occupational stressors and, thus, result in an under – rather than an over – estimation of the association between psychosocial job dimensions and distress/well-being variables.

Despite these limitations, the results of this study show that the individual construct OCSE is both directly associated with employee distress/well-being and plays a moderating role in the relationship between psychosocial job characteristics and distress/well-being. Further examination of the role of OCSE seems essential to enhance our understanding of the impact of job characteristics on employee distress.

## Conflict of Interest Statement

The authors declare that the research was conducted in the absence of any commercial or financial relationships that could be construed as a potential conflict of interest.
